# Subgrain Size Modeling and Substructure Evolution in an AA1050 Aluminum Alloy during High-Temperature Compression

**DOI:** 10.3390/ma17174385

**Published:** 2024-09-05

**Authors:** Qi Yang, Tomasz Wojcik, Ernst Kozeschnik

**Affiliations:** Institute of Materials Science and Technology, TU Wien, Getreidemarkt 9, 1060 Vienna, Austria; yangqi.tuwien@outlook.com (Q.Y.);

**Keywords:** substructure evolution, subgrain size model, high-temperature compression, aluminum alloy

## Abstract

For materials with high stacking fault energy (SFE), such as aluminum alloys, dynamic recovery (DRV) and dynamic recrystallization (DRX) are essential softening mechanisms during plastic deformation, which lead to the continuous generation and refinement of newborn subgrains (2° ˂ misorientation angle ˂ 15°). The present work investigates the influence of compression parameters on the evolution of the substructures for a 1050 aluminum alloy at elevated temperatures. The alloy microstructure was investigated under deformation temperatures ranging from 300 °C to 500 °C and strain rates from 0.01 to 0.1 s^−1^, respectively. A well-defined substructure and subsequent subgrain refinement provided indication of the evolution laws of the substructure under high-temperature compression. Corresponding experimental data on the average subgrain size under various compression conditions were obtained. Two different independent average subgrain size evolution models (empirical and substructure-based) were used and applied with several internal state variables. The substructure model employed physical variables to simulate subgrain refinement and thermal coarsening during deformation, incorporating a corresponding dislocation density evolution model. The correlation coefficient (*R*) and root mean square error (*RMSE*) of the substructure-based model were calculated to be 0.98 and 5.7%, respectively. These models can provide good estimates of the average subgrain size, with both predictions and experiments reproducing the expected subgrain size evolution using physically meaningful variables during continuous deformation.

## 1. Introduction

Aluminum alloys are widely used in automotive, aerospace, and other branches of industry due to their low mass and mechanical features. The hot forming of Al alloys is a complex metallurgical process that involves work hardening (WH), dynamic recovery (DRV), static recovery (SRV), dynamic recrystallization (DRX), grain growth, and other phenomena [1,2,3]. 

For materials with high stacking fault energy (SFE), such as Al alloys, DRV and continuous dynamic recrystallization (CDRX) [4,5,6,7,8] are the prominent softening mechanisms during hot forming, and they are observed in different series of Al alloys. Recent research [9,10,11] has indicated that low-angle subgrains (2° < misorientation angle < 15°) develop through the absorption of new mobile dislocations, subsequently transforming into high-angle grain boundaries (HAGBs) with misorientation angles > 15°. This process results in a well-defined substructure and subsequent DRX during high-temperature deformation. 

The understanding and modeling of low-angle subgrain evolution are recognized as the key for describing the mechanisms of DRV and DRX during plastic deformation. Huang et al. [12] and Sakai et al. [13] reviewed the microstructural evolution and mechanism of Al alloy during deformation. Zhang et al. [14], Ding et al. [15], and Li et al. [16] studied the deformation behavior and DRX of various Al alloys, such as 2195 Al alloy and 5083 Al alloy. Current research has focused more on the CDRX process for Al alloy, which has been observed in the hot deformation or severe plastic deformation (SPD) processes of Al-Mg [17], Al-Li [18], Al-Cu [19], and other Al alloys. There are only a few studies that have analyzed and investigated substructural changes under varying compression conditions.

The majority of existing models [20,21,22,23] rely on empirical relationships to quantify parameters such as flow stress and steady-state subgrain size with the Zener–Hollomon parameter (*Z*). Material models of the subgrain size evolution of high-SFE alloy are scarcer. Nes et al. [24,25] as well as Duan and Sheppard [26,27,28] have developed mathematical models to describe the substructure evolution and the recrystallization kinetics during aluminum rolling and extrusion, respectively. In subsequent work, Marthinsen and Nes [29] considered the influence of grains, particles, and dispersoids on the subgrain size model. Gourdet and Montheillet [30] (GM) introduced a subgrain size evolution model as part of the CDRX model framework, which considers the misorientation evolution caused by subgrain rotation and the migration of HAGBs. Furthermore, some extended subgrain models [31,32,33] have also been developed recently on the basis of the GM model within the CDRX framework. 

Most of the subgrain models developed in recent years rely on translating subgrain size $\delta_{sub}$ into volumetric boundary density $S_{sub}$, defined as $\delta_{sub}=2/S_{sub}$ [30,31,32,33]. This approach lacks physical interpretability and is challenging to verify experimentally. Currently, subgrain size can be measured experimentally using electron backscatter diffraction (EBSD), allowing for the investigation of its effects on material properties. An independent model of average subgrain size, incorporating physically meaningful variables, is applied based on experimental values. In this study, an AA1050 pure Al alloy was selected to minimize the influence of solute atoms and precipitated particles as defects/pinning position on the evolution of low-angle subgrain boundaries (or dislocation cells).

In this study, the influence of deformation parameters on the evolution of the substructures for an AA1050 Al alloy was investigated under high-temperature compression, experimentally obtaining the average grain size under various conditions. The occurrence of a well-defined substructure and subsequent low-angle subgrain refinement were analyzed under different strains, temperatures, and strain rates. The mechanisms driving substructure evolution were evaluated by analyzing the average subgrain size, misorientation angles, and the distribution of subgrains. Subsequently, an empirical model and an advanced substructure-based model of average subgrain size were established with several internal state variables. These models were validated using a detailed substructural investigation, and model input parameters are herein described in detail as well as their dependencies.

## 2. Model Description

### 2.1. Empirical Average Subgrain Size Model

During the hot forming of Al alloys, DRV serves as the main recovery process, resulting in the formation of well-defined substructures. Recent studies have indicated that the average subgrain size gradually attains a “saturation value” $\delta_{s}$, which becomes invariant with respect to strain during deformation [1,21,22,23,24,34]. This saturated subgrain size is balanced during DRV after the alloy microstructure reaches steady state, and it depends on the temperature and strain rate, as in [21].

(1) $$\delta_{s}={\left[\alpha_{1}+\beta_{1}\ln\left(\dot{\varepsilon }exp(\frac{Q}{RT})\right)\right]}^{-1}$$

where $\delta_{s}$ is the saturated average subgrain size, $\alpha_{1}$ and $\beta_{1}$ are material constants, $\dot{\varepsilon }$ is the strain rate, Q is the deformation activation energy, *R* is the gas constant, and *T* is the absolute deformation temperature. In the present work, the values of $\alpha_{1}$ and $\beta_{1}$ are derived from the EBSD experimental data, with the average value of the coefficients being $\alpha_{1}$ = 0.015 and $\beta_{1}$ = −0.15.

The activation energy *Q* is a key material parameter that indicates the resistance of the deformation. In the present work, the activation energy *Q* is 142 kJ.mol^−1^, close to that for pure Al alloy [35].

The empirical model describes the process of subgrain evolution under deformation based on the initial subgrain size $\delta_{0}$, which gradually decreases until a steady-state microstructure is achieved. The relationship between the average subgrain size and the saturated subgrain size is represented by the following empirical formulation [36]:

(2) $$\left\{\begin{array}{c} \delta_{sub}=\delta_{0}-\left(\delta_{0}-\delta_{s}\right)\left[1-\exp\left(-\alpha_{2}{\left(\varepsilon_{d}-\varepsilon_{c}\right)}^{\beta_{2}}\right)\right],\;\;\;\;\varepsilon_{d}<\varepsilon_{s}\\ \delta_{sub}=\delta_{s},\;\;\;\;\;\;\varepsilon_{d}>\varepsilon_{s} \end{array}\right.$$

where $\delta_{sub}$ is the average subgrain size, $\delta_{0}$ is the initial average subgrain size, $\alpha_{2}$ and $\beta_{2}$ are material constants, $\varepsilon_{d}$ is the strain during deformation, $\varepsilon_{c}$ is the critical strain up to which subgrains can be distinguished, and $\varepsilon_{s}$ is the strain where the subgrain size starts to become saturated.

To determine the values of $\alpha_{2}$ and $\beta_{2}$, Equation (2) can be rewritten in double natural logarithm form:

(3) $$\ln\left(-\ln\left(1-\frac{\delta_{0}-\delta_{sub}}{\delta_{0}-\delta_{s}}\right)\right)=\ln\alpha_{2}+\beta_{2}\ln(\left(\varepsilon_{d}-\varepsilon_{c}\right))$$

In the present work, the material constants $\alpha_{2}$ and $\beta_{2}$ are derived from the mean values of the slopes and intercepts as $\alpha_{1}$ = 5.00 and $\beta_{1}$ = 1.15.

### 2.2. Substructure-Based Model

The plastic deformation of Al alloys generally occurs in three key stages [1,12,13,25]: (1) initial stage: increasing dislocation activity, whereby cells/subgrain boundaries are formed; (2) subgrain refinement: subgrains continue to form and refine; and (3) thermal coarsening of subgrain: involves the thermally induced coarsening of subgrains during deformation, resulting in an increase in subgrain size.

The substructure-based model of average subgrain size describes the evolution of subgrains using physical internal state variables. The first part of the model is to simulate the subgrain refinement as the deformation progresses, incorporating the effects of various deformation parameters. To better represent the subgrain refinement process during high-temperature compression, a substructure-based model was developed as follows [25,29]:

(4) $$d\delta_{sub}^{-}=-f_{r}\frac{\sqrt{3}\dot{\varepsilon }b^{1/2}}{{{\bar{\theta }}_{s}}^{3/2}{{\bar{\delta }}_{s}}^{2}}{\delta_{sub}}^{v_{1}}dt$$

where $\delta_{sub}$ is the average subgrain size during deformation, *b* is the Burger’s vector, ${\bar{\theta }}_{s}$ is the average subgrain boundary misorientation in the steady-state stage, $v_{1}$ is a material coefficient that controls the subgrain refinement rate and is set to 1 in this work, $f_{r}$ is a material coefficient related to subgrain refinement, and ${\bar{\delta }}_{s}$ is the average subgrain size of steady-state stage, expressed as follows:

(5) $${\bar{\delta }}_{s}=\frac{1}{3}{\left(\frac{{\bar{\sigma }}_{s}}{c_{1}Gb}\right)}^{-1},\;\mathrm{with}\;{\bar{\sigma }}_{s}=f_{1}[{\dot{\varepsilon }exp(\frac{Q}{RT})]}^{f_{2}}$$

where ${\bar{\sigma }}_{s}$ is the steady-state flow stress, G is the shear modulus, $f_{1}$ and $f_{2}$ are material constants, and $c_{1}$ is a material coefficient related to the flow stress.

In the process of high-temperature compression, the occurrence of subgrain refinement is dynamically counteracted by thermally induced coarsening. This coarsening occurs by mechanisms such as subgrain boundary migration, subgrain rotation, and the evolution of dislocation density, resulting in the movement of subgrain boundaries. In the present work, a coarsening model was applied, where the subgrain size is related to the evolution of subgrain migration and dislocation density, as follows [25]:

(6) $$d\delta_{sub}^{+}=f_{c}\nu_{D}b^{2}\sqrt{\rho }\left[\exp\left(-\frac{U_{s}+PV_{a}}{k_{B}T}\right)\right]dt$$

where $\nu_{D}$ is the Debye frequency, ρ is the dislocation density, P is the driving force on the subgrain boundary, $V_{a}$ is the average activation volume of subgrain boundaries, $k_{B}$ is the Boltzmann constant, $U_{s}$ is the activation energy for self-diffusion in Al with unit J.atom^−1^, and $f_{c}$ is a material coefficient related to subgrain coarsening.

For the two material coefficients, the coefficient $f_{r}$ exhibits sensitivity to both deformation temperature and strain rate, making it a critical factor in controlling the trend and rate of subgrain refinement under various deformation conditions. Similarly, the material coefficient $f_{c}$ is also dependent on temperature and strain rate. Therefore, the coefficients can be formulated as given below:

(7) $$f_{r}=f_{3}[{\dot{\varepsilon }exp(\frac{Q}{RT})]}^{f_{4}},\;\mathrm{and}\;f_{c}=f_{5}[{\dot{\varepsilon }exp(\frac{Q}{RT})]}^{f_{6}},$$

where $f_{3}$, $f_{4}$, $f_{5}$, and $f_{6}$ are material constants.

The driving force for subgrain boundary migration [37] is taken as $P={4\gamma /\delta }_{sub}$, where γ is the subgrain boundary energy. The average activation volume of subgrain boundary [25] can be expressed as $V_{a}=b^{3}/\theta_{sub}$, where $\theta_{sub}$ is the average subgrain boundary misorientation angle.

The dislocation density model incorporates several key mechanisms affecting the dislocation evolution, including dislocation generation due to work hardening (WH) and reduction in dislocation density through DRV and static recovery (SRV) with dislocation climbing and gliding, as follows [38,39,40]:

(8) $$d\rho /dt=\frac{M\sqrt{\rho }}{Ab}\dot{\varepsilon }-2BM\rho \frac{d_{crit}}{b}\dot{\varepsilon }-2CD_{d}\frac{Gb^{3}}{k_{b}T}\left(\rho^{2}-{\rho_{eq}}^{2}\right)dt-2f_{H}v_{H}\rho \frac{1}{\delta_{sub}}$$

where *A*, *B*, and *C* are material-dependent coefficients; M is the Taylor factor; $d_{crit}$ is the critical distance of dislocation annihilation; $D_{d}$ is the diffusion coefficient along dislocation pipes; $\rho_{eq}$ is the equilibrium dislocation density; $f_{H}$ is the fraction of HAGBs; and $v_{H}$ is the migration rate of HAGBs.

The last term in Equation (8) accounts for the reduction in the average internal dislocation density accompanying the migration of HAGBs. The migration rate of HAGBs is adopted from Gourdet and Montheillet [30] as follows:

(9) $$v_{H}=v_{0}{\left(\frac{\dot{\varepsilon }}{\dot{\varepsilon_{0}}}\right)}^{m},$$

where $\dot{\varepsilon_{0}}$ is the initial strain rate, $v_{0}$ is the initial migration rate of HAGBs, and m is a material constant related to the migration rate.

## 3. Experimental Results

### 3.1. Material and Experiments

**Figure 1:**
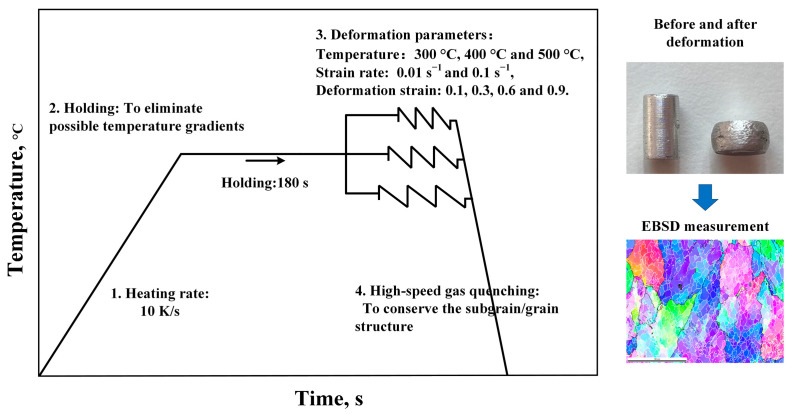
Schematic of the isothermal compression experiments history.

In this study, AA1050 Al alloy billets provided by Neuman Aluminium Austria GmbH (Marktl, Austria) were investigated and subsequently machined into cylinders with a diameter of 5 mm and a height of 10 mm. Single-pass isothermal compression was conducted using a deformation dilatometer DIL 805 A/D from Bähr (Hüllhorst, Germany). The procedures of the compression experiments are shown in Figure 1. Three different forming temperatures within the range of 0.5*T*_m_ < *T*_def_ < 0.7*T*_m_ [11] (300 °C, 400 °C, and 500 °C), two different strain rates (0.01 s^−1^ and 0.1 s^−1^), and four true strains (0.1, 0.3, 0.6, and 0.9) were applied. The true strain values were set within the deformation dilatometer using a built-in calculation formula, defined as $true\;strain=Ln(L/L_{0})$, where $L_{0}$ is the original length of samples, and L is the current length after deformation. The specimens were quenched with over 100 K/s cooling speed to room temperature immediately after compression.

EBSD tests were carried out in a Zeiss Sigma 500 VP (Oberkochen, DE) high-resolution scanning electron microscope with an EDAX detector with an accelerating voltage of 20 kV and a step size of 0.5 μm. The data processing of EBSD results was carried out with the EDAX OIM Analysis8 software (v8, EDAX Inc., Mahwah, NJ, USA). The post-processing procedure to distinguish most of the subgrains consisted of the following: (1) the standard “clean up” algorithm; (2) construction of the grains with grain tolerance angle 1.0, which enabled the software to identify most subgrains; (3) setting up a confidence index ˃ 0.1 filter in the partition properties dialog to hide all mis-indexed points; (4) performing a confidence index standardization (CIS) to allow points from overlapping patterns at grain boundaries to be maintained in the map; (5) acquiring distinct grain color maps and subgrain size distributions, and areas containing significant errors or unresolved regions were excluded from the analysis.

### 3.2. Substructure Evolution

**Figure 2:**
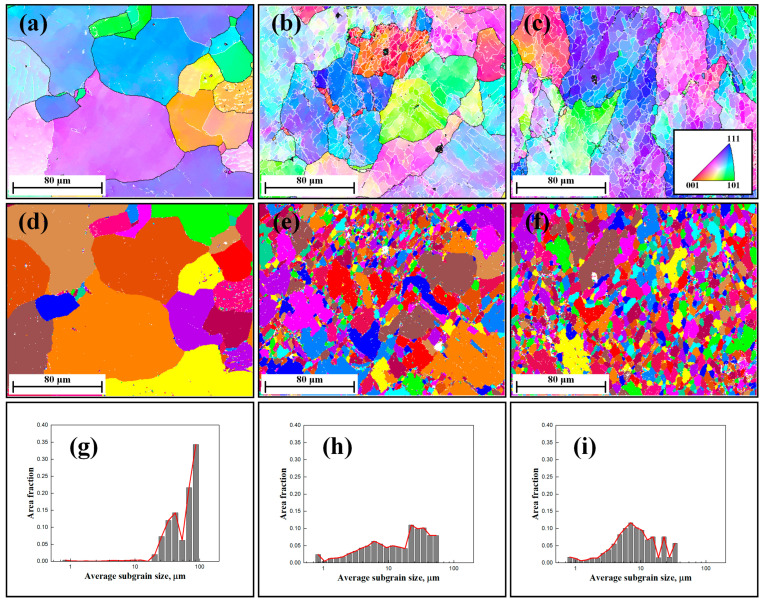
EBSD images of 1050 Al alloy deformed to a different strain at a temperature of 300 °C and a strain rate of 0.1 s^−1^: (**a**–**c**) inverse pole figure (IPF) maps of the specimens with strain 0.1, 0.3, and 0.6; (**d**–**f**) corresponding unique grain color maps; and (**g**–**i**) subgrain size distribution maps.

Figure 2a–c show the microstructure maps after compression at temperatures of 300 °C. Low-angle subgrain boundaries and HAGBs are shown as white and black lines, respectively. Under conditions of low strain (Figure 2a), the generation of initial subgrains was observed within the parent grains, characterized by an irregular morphology. In materials with high SFE, dislocations rearrangement and annihilation occurred through DRV, resulting in the formation of subgrain boundaries within the pancaked parent grains [12,13,21,22]. It is noteworthy that the initial subgrain boundaries were formed without a nucleation process, consistent with the conclusions reported by Huang et al. [12] and Sakai et al. [13].

As deformation progressed (Figure 2b), an increased formation of subgrain boundaries was observed due to the continued accumulation of dislocations. Concurrently, the generation of some unclosed subgrains within the original grains was observed, which is also shown by the corresponding unique grain color maps depicted in Figure 2e. Additionally, Figure 2c reveals the presence of more newborn subgrains with an equiaxed morphology, suggesting subgrain rotation and an increase in the misorientation angle of boundaries. This continuous formation of new subgrain boundaries resulted in a substantial decrease in the average subgrain size.

The corresponding unique grain color maps and subgrain size distribution maps at 300 °C are presented in Figure 2d–i. It is clear that the average subgrain size (area fraction) exhibits a relatively random distribution. The average subgrain size of the specimens progressively decreased with increasing strain, decreasing deformation temperature, and increasing strain rate. Consequently, the continuous formation of subgrain boundaries was responsible for the decrease in the subgrain size, which was also accompanied by an increase in wall dislocation density and misorientation angle, serving as a typical identification of DRV and, subsequently, the CDRX phenomenon. [1,12,13] The average subgrain size, calculated from the EBSD data under different conditions, is summarized in Table 1.

**Figure 3:**
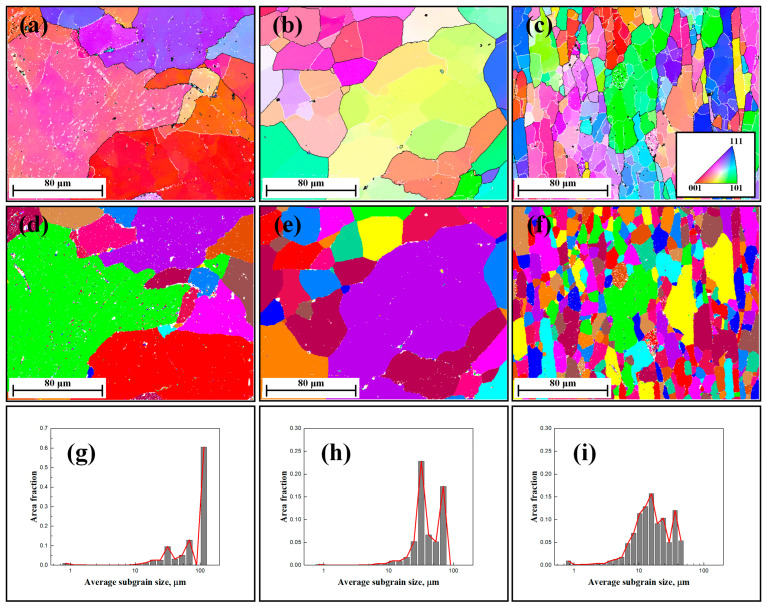
EBSD images of 1050 Al alloy deformed to a different strain with a temperature of 500 °C and a strain rate of 0.1 s^−1^: (**a**–**c**) IPF maps of the specimens deformed with strain 0.1, 0.3, and 0.6; (**d**–**f**) corresponding unique grain color maps; and (**g**–**i**) subgrain size distribution maps.

Different from Figure 2, the presence of subgrain coarsening became evident when subjected to deformation at high temperatures, as shown in Figure 3b,c. During the DRV process, elevated temperatures enhanced their mobility, facilitating the migration of subgrain boundaries and thereby leading to an increase in subgrain size [25,29]. As deformation proceeded, the primary mechanism remained the continued generation and refinement of finer subgrains, which was manifested in a significant reduction in subgrain size, as shown in Figure 3d–i and Table 1. This demonstrates that some low-angle sub-boundaries disappeared, while others expanded due to the continuous growth of subgrains (cells) during hot deformation [41]. Consequently, the increasing of mean subgrain misorientation remained constant even under high strain conditions, in agreement with results from McQueen and Kassner [42].

**Figure 4:**
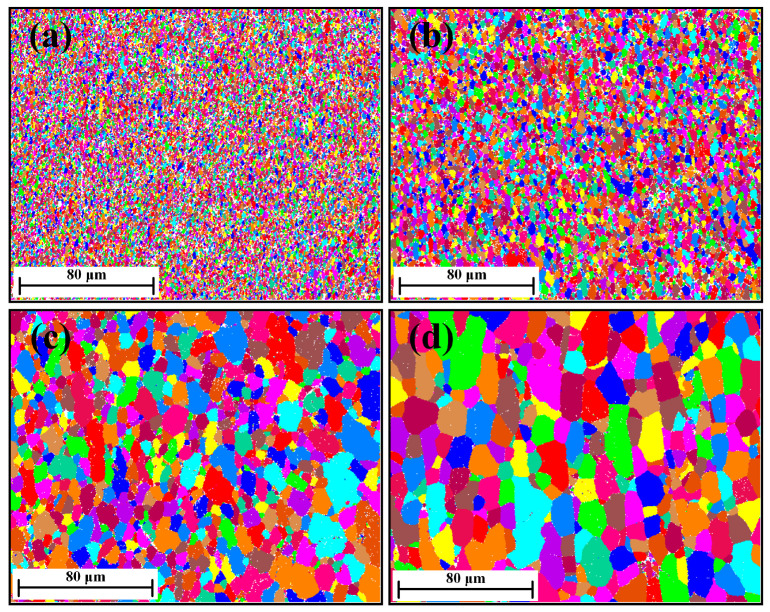
Unique grain color maps of 1050 Al alloy deformed to strain 0.9 at different deformation conditions: (**a**) 300 °C/0.1 s^−1^, (**b**) 400 °C/0.1 s^−1^, (**c**) 500 °C/0.1 s^−1^, and (**d**) 500 °C/0.01 s^−1^.

The unique grain color maps of specimens deformed to strain 0.9 are illustrated in Figure 4. As expected, the average subgrain size was sensitive to both deformation temperature and strain rate; i.e., the subgrain size increased with increasing temperature or decreasing strain rate.

## 4. Model Application and Discussion

### 4.1. Model Input Parameters

The initial parameters for the variables are defined as follows: The initial average subgrain size $\delta_{0}$ was 100 μm. Furthermore, the critical strain $\varepsilon_{c}$, at which subgrain boundaries become distinguishable within the deformed microstructure, was simplified to zero.

The average subgrain boundary misorientation ${\bar{\theta }}_{s}$ of the steady-state stage can be found in the literature as 3°, as suggested by Nes [25]. The value of the material coefficient $c_{1}$ was 60, and the calculation of ${\bar{\sigma }}_{s}$ was temperature- and strain rate-dependent, such as ${\bar{\sigma }}_{s}=0.6[{\dot{\varepsilon }exp(Q/RT)]}^{0.16}$ MPa. The value of subgrain boundary energy γ was selected to be 0.3 J.m^−2^ in this work [37], and the activation energy for self-diffusion in Al [43] was 2·10^−19^ J.atom^−1^. The list of input parameters for the substructure-based model is summarized in Table 2.

The dislocation density evolution parameters *A*, *B*, and *C* were adjusted to the experimental flow curve with the empirical coefficient a in the Taylor relationship, taken as 0.2. The calculation of the critical distance for dislocation annihilation $d_{crit}$ and diffusion coefficient along dislocation pipes $D_{d}$ can be found in the literature as suggested by Sherstnev, Lang, and Kozeschnik [38] and Kreyca and Kozeschnik [39]. The parameters of the HAGBs migration rate were selected for Al alloy as suggested by Gourdet and Montheillet [30].

### 4.2. Model Validation

This section presents a comparative analysis of the simulated results from the substructure-based model against the experimental data on the evolution of average subgrain size. The experimental measurements of average subgrain size were obtained by EBSD and are detailed in Table 1. The simulations were conducted using MATLAB software version R2016b with given true strain values, employing one single set of input parameters (refer to Section 4.1) applied across all deformation conditions.

**Figure 5:**
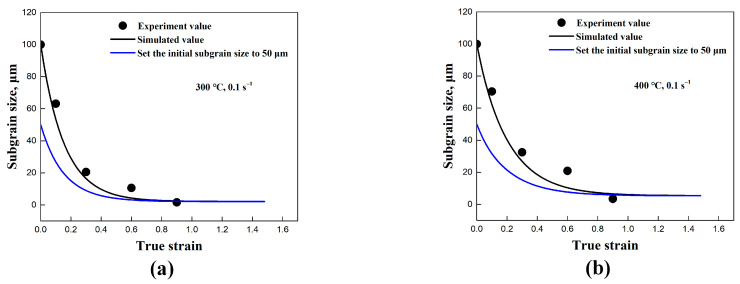

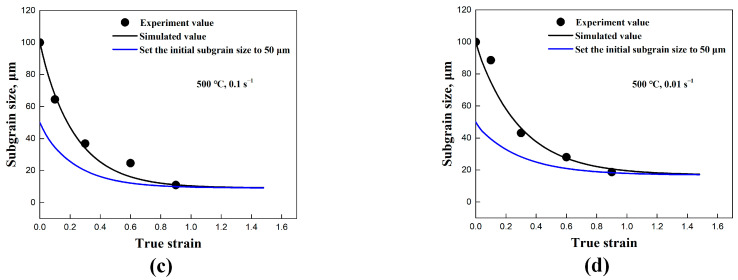
The comparison between the experimental and simulated values of the substructure-based model with different deformation conditions of (**a**) 300 °C/0.1 s^−1^, (**b**) 400 °C/0.1 s^−1^, (**c**) 500 °C/0.1 s^−1^, and (**d**) 500 °C/0.01 s^−1^.

Figure 5 presents a comparison between the substructure-based model and the experimental values. At the onset of deformation, a substantial formation of new subgrain boundaries occurred with the progression of compression, resulting in a rapid reduction in the average subgrain size. As the compression progressed, the rate of subgrain size reduction decreased. It is suggested that the subgrain size eventually reaches a “saturation value” at higher strain [1,21,22,23,24,34]. The analysis indicated that the substructure-based model clearly reproduced the evolution of the subgrain size. Both the model and the experimental data exhibited the anticipated behavior of the average subgrain size evolution during continuous deformation. Furthermore, this model’s ability to simulate subgrain size evolution from different initial average subgrain sizes (50 μm) demonstrates its applicability under various initial subgrain sizes.

To further evaluate the performance of the substructure-based model in the present study, the correlation coefficient (*R*) and root mean square error (*RMSE*) [50] were evaluated as follows:

(10) $$R=\frac{\sum_{i=1}^{N}\left(\delta_{ei}-{\bar{\delta }}_{e})(\delta_{ci}-{\bar{\delta }}_{c}\right)}{\sqrt{\sum_{i=1}^{N}{\left(\delta_{ei}-{\bar{\delta }}_{e}\right)}^{2}\sum_{i=1}^{N}{\left(\delta_{ci}-{\bar{\delta }}_{c}\right)}^{2}}}$$

(11) $$RMSE=\sqrt{\frac{1}{N}\sum_{i=1}^{N}{{(\delta }_{ci}-\delta_{ei})}^{2}}\cdot 100\%$$

where $\delta_{ci}$ represents the calculated subgrain size, $\delta_{ei}$ represents the experimental subgrain size, ${\bar{\delta }}_{c}$ is the average calculated subgrain size, ${\bar{\delta }}_{e}$ is the average experimental subgrain size, and *N* is the total number of data points used in this study.

**Figure 6:**
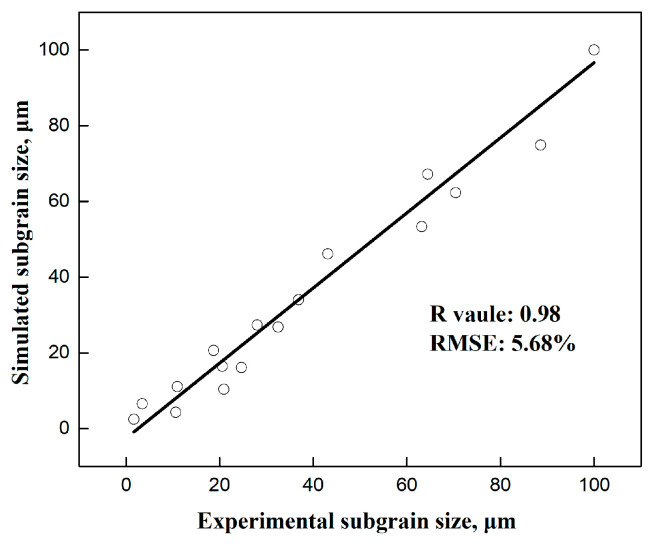
Error analysis for the substructure-based model.

The corresponding error analysis for different deformation conditions is illustrated in Figure 6. The values of *R* and *RMSE* are 0.98 and 5.68%, respectively, indicating a good agreement between the experiment and the model.

**Figure 7:**
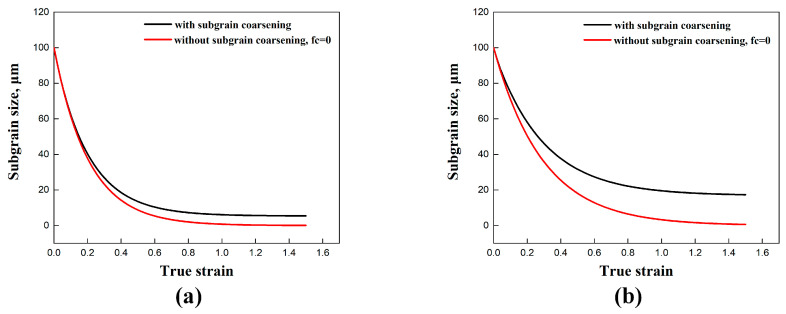
Illustration of the subgrain size evolution with material coefficients related to subgrain refinement and coarsening: (**a**) 400 °C/0.1 s^−1^ and (**b**) 500 °C/0.01 s^−1^.

**Figure 8:**
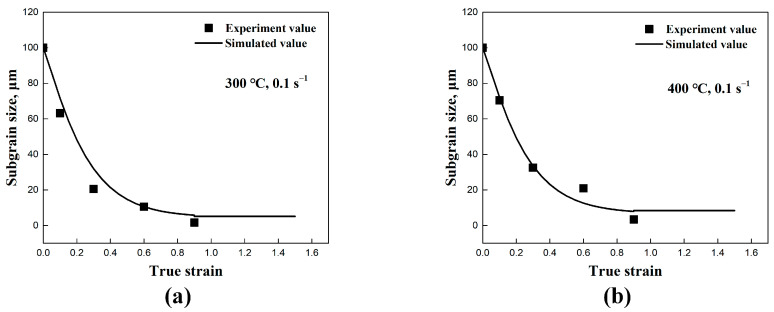

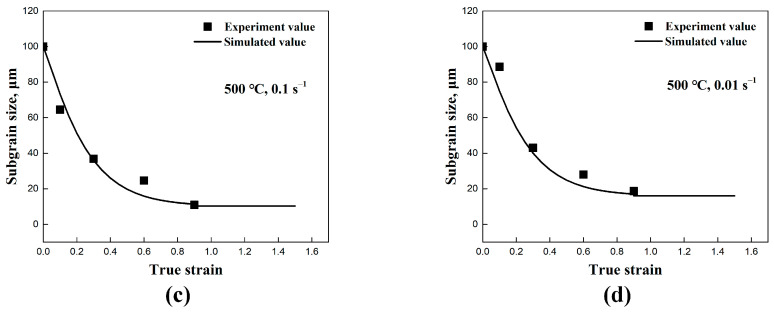
The comparison between the simulated average subgrain size $\delta_{sub}$ and experimental $\delta_{sub}$ of the empirical model: (**a**) 300 °C/0.1 s^−1^, (**b**) 400 °C/0.1 s^−1^, (**c**) 500 °C/0.1 s^−1^, and (**d**) 500 °C/0.01 s^−1^.

In the substructure-based model, the material coefficients $f_{r}$ and $f_{c}$ directly affect the subgrain refinement and coarsening, as illustrated in Figure 7. By accounting for the coarsening term, the refinement of the initial subgrain size is clearly observed, as demonstrated in the microstructures shown in Figure 2 and Figure 3. The subgrain size decreased and gradually reached a steady-state value at larger strains, in agreement with studies from Sellars et al. [22] and Furu et al. [23]. In contrast, when subgrain coarsening was not considered (with the coefficient $f_{c}$ set to 0), a steady state trend was not achieved, and the subgrain size continued to decrease until it reached zero. Correspondingly, the continuous generation of new subgrain boundaries through dislocation accumulations and rearrangements was observed, in agreement with the results of subgrain/grain boundary migration from Gourdet and Montheillet [30]. In addition, the comparison between the simulated average subgrain size $\delta_{sub}$ and experimental $\delta_{sub}$ of the traditional empirical model is also shown in Figure 8.

### 4.3. Discussion

**Figure 9:**
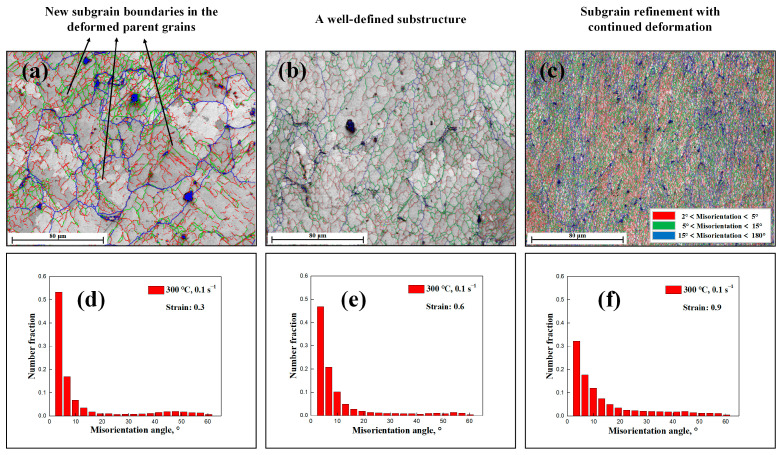
EBSD images of 1050 Al alloy deformed to a different strain at a temperature of 300 °C and a strain rate of 0.1 s^−1^: (**a**–**c**) grain boundary maps of the specimens deformed with strain 0.3, 0.6, and 0.9 and (**d**–**f**) corresponding distribution histograms of misorientation angle.

The analysis of the deformation structure from the EBSD results confirms that hot deformation leads to the formation of a substructure, where the density of low-angle subgrain boundaries continuously increases (see Figure 2 and Figure 3). Figure 9 represents a detailed grain boundary maps to facilitate a better understanding of substructure generation and development. The formation of cell boundaries, characterized by a high density of dislocations, is constituted by geometrically necessary dislocations (GNDs) that serve to divide the parent grains into subgrains and to maintain the deformation gradients inside the grain as well as the rotation of subgrains. In the deformed microstructure at the interrupted strain of 0.3 (Figure 9a), a high fraction of incomplete sub-boundaries (2° ˂ misorientation ˂ 15°) indicates the formation of a dislocation cell structure. Due to DRV, these incomplete low-angle subgrains (cell walls) continue to form fine and polygonized substructures with ongoing deformation. This mechanism is observed in Al alloys [4,8,19,51], 304-type austenitic stainless steel [52], Ti alloy [53], etc.

In addition, some researchers [8,12,13] have introduced other mechanisms for subgrain boundary formation, including micro-shear band assistance and progressive lattice rotation near grain boundaries. At elevated deformation temperatures (see Figure 3), a homogeneous microstructure generally forms, and deformation/micro-shear bands become less prominent compared to their presence at lower temperatures or cold deformation. The formation of subgrain boundaries facilitated by micro-shear bands is typically observed during the SPD process [13].

Figure 9d–f display the misorientation angle histograms with different strains at 300 °C/0.1 s^−1^, which indicate a relatively random distribution of misorientation angle. Notably, the average misorientation angle of the specimens increases progressively with strain. This increase is attributed to the transformation of low-angle subgrain boundaries into HAGBs with absorbing mobile dislocations. Such transformations are a feature of the CDRX mechanism, in accordance with the conclusions of other researchers [1,7,8,9,10,11,12,13,14].

**Figure 10:**
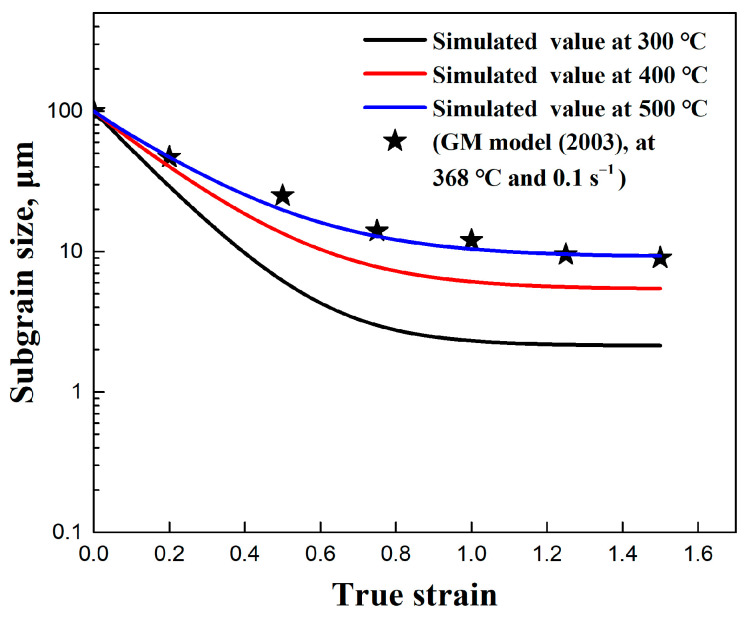
Simulated average subgrain size ($\delta_{sub}$) by substructure-based model with different temperatures at a strain rate 0.1 s^−1^ and comparing the simulated value with the result from Gourdet and Montheillet (GM model) [27].

In the substructure-based model, the objective is to correlate the subgrain size evolution with deformation thermo-mechanics, utilizing several internal state variables. Suitable parameters are selected under different strain, strain rate, and temperature to reproduce the observed evolution of average subgrain sizes across the spectrum of conditions (see Section 4.1). The effect of deformation conditions (temperature and strain rate) on the microstructure is shown in Figure 2, Figure 3 and Figure 4. During deformation at high temperatures, coarse subgrains become evident. Figure 10 shows the simulated average subgrain size predicted by the substructure-based model under different temperatures at a strain rate of 0.1 s^−1^. An increase in temperature promotes the generation of subgrain boundaries and their mobility, thus promoting the formation of DRX grains [12,13]. A similar observation was reported for the GM model in a 1200-grade aluminum alloy from Gourdet and Montheillet [30], with the differences compared to our present results most likely being due to the differences in the alloy composition. Qualitatively, the results are in reasonable agreement.

**Figure 11:**
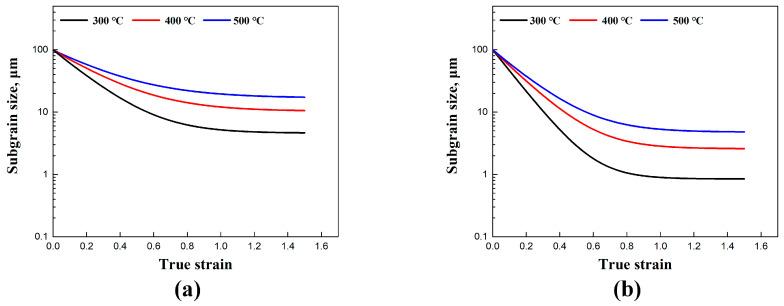
Simulated average subgrain size ($\delta_{sub}$) by the substructure-based model with different temperatures at a strain rate of (**a**) 0.01 s^−1^ and (**b**) 0.8 s^−1^.

The simulated results of average subgrain size at other deformation conditions are shown in Figure 11. An increase in strain rate increases the dislocation density, promoting the formation of subgrain boundaries [54,55]. Additionally, the deformation time is elongated at a lower strain rate, facilitating the migration of subgrain boundaries, which in turn increases the subgrain size [11,12,13]. The results from the experimental subgrain size curves show that the refinement rate is initially high during the early stages of compression but subsequently decelerates. This phenomenon is well simulated by the applied models after the incorporation of thermal subgrain coarsening, in agreement with the results from Nes [25], Gourdet and Montheillet [30], Maizza et al. [10], Sun et al. [31], and Chen et al. [32].

The substantial generation of new subgrains significantly influences the mechanical properties of the material. The correlation between mechanical properties and microstructure is discussed in the context of subgrain strengthening. Typically, subgrain boundary strengthening is considered to be inversely proportional to the square of the average subgrain size, as described by the Hall–Petch relationship (case 1) [56],

(12) $$\sigma_{sub}=k_{sub}{\delta_{sub}}^{-1/2},$$

where $\sigma_{sub}$ is the strengthening contribution coming from subgrain boundaries, and $k_{sub}$ is a material constant for subgrain boundary strengthening, set as 10 MPa·μm^1/2^. In this work, the value of $\delta_{sub}$ was calculated using the substructure-based model.

Another subgrain strengthening model (case 2) was used to compare with the empirical Hall–Petch relationship, as suggested by Marthinsen and Nes [29]. The formula can be written as follows:

(13) $$\sigma_{sub}=\alpha_{sub}MGb/\frac{b}{\delta_{sub}}$$

where $\alpha_{sub}$ is a material constant and was selected to be 0.83 for Al alloy, as suggested by Duan and Sheppard [27].

**Figure 12:**
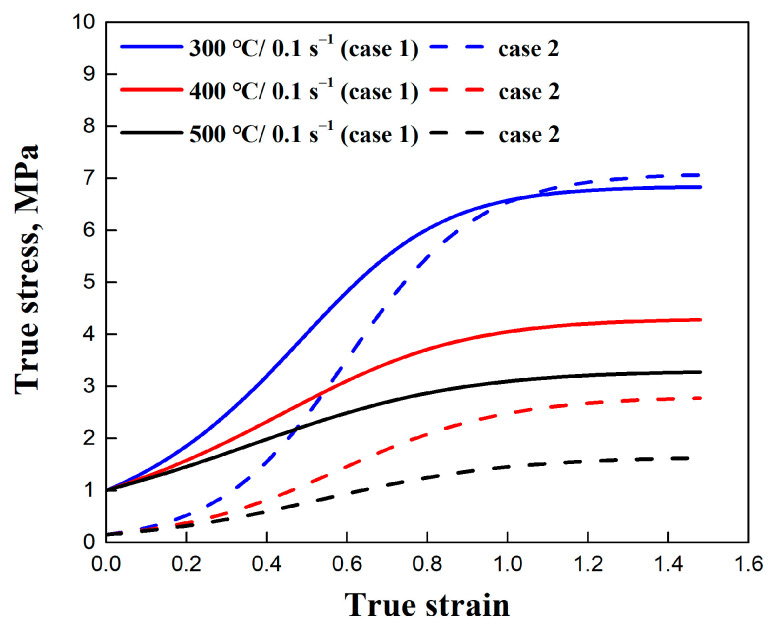
Subgrain strengthening calculations of Hall–Petch relationship (case 1) and Marthinsen and Nes model (case 2) under different deformation conditions.

Figure 12 illustrates the subgrain strengthening calculations of the Hall–Petch relationship (case 1) and Marthinsen and Nes model (case 2) under different deformation conditions. The refinement of subgrain results in strength improvements and their corresponding characteristics. During the initial stage of deformation, the subgrain size is relatively large, leading to minimal subgrain boundary strengthening. As deformation progresses, the generation of a significant number of fine subgrains contributes to an increase in stress. Once the subgrain size exceeds the critical threshold for refinement, the steady-state subgrain size results in a balanced rate of increase in subgrain strengthening, in agreement with studies from Hansen [56] and Summers et al. [57].

## 5. Conclusions

The paper reflects the substructure evolution of an AA1050 Al alloy under various deformation conditions during high-temperature compression. Subsequently, two subgrain size evolution models were applied based on EBSD experiments. The main conclusions are as follows:

(1) A detailed substructure is provided with subgrain/grain boundary characteristics. The results revealed the dependence of the average subgrain size on factors such as temperature, strain rate, and strain, and their special roles on subgrain refinement and thermal coarsening were captured;

(2) The mechanism and deformation variables, including subgrain formation and refinement, were discussed in detail, considering the average subgrain size, misorientations angles, and the distribution of subgrains. The coexistence of subgrain refinement and thermal coarsening varies with changes in deformation conditions and the corresponding average subgrain size was also measured directly from EBSD experiments;

(3) An empirical model and an advanced substructure-based model of average subgrain size were established with several internal state variables. In the established models, the process of subgrain evolution is described by introducing saturated average subgrain size, dislocation density, misorientation angle, subgrain boundary energy, etc. The evolution of average subgrain size can be effectively well reproduced across a range of temperatures and strain rates;

(4) In the substructure-based model, various factors such as the material coefficients, initial subgrain size, temperature, and strain rate were analyzed. The correlation coefficient (*R*) and root mean square error (*RMSE*) of the substructure-based model were calculated to be 0.98 and 5.7%, respectively, which indicate good agreement between the experiment and the model;

(5) The mechanism of subgrain boundary formation and refinement were discussed in detail, and subgrain strengthening was also studied with the Hall–Petch relationship.

## Figures and Tables

**Table 1:** Measured average subgrain size under different deformation conditions.

Average Subgrain Size (μm)	Temperature (°C)	Strain Rate (s^−1^)	Strain 0.1	Strain 0.3	Strain 0.6	Strain 0.9
subgrain size	300	0.1	63	21	11	1.7
	400	0.1	71	33	21	3.5
	500	0.1	65	37	25	11
	500	0.01	83	43	28	19

**Table 2:** List of input parameters for substructure-based model.

Symbol	Name	Unit	Value	Ref.
ν	Poisson’s ratio	-	0.347	[44]
G	Shear modulus	MPa	29,438.4–15.052*T*	[45,46]
b	Burgers vector	m	2.86·10^−10^	[47]
M	Taylor factor	-	3.06	[48]
$\nu_{D}$	Debye frequency	s^−1^	1·10^13^	[49]
$\theta_{sub}$	Subgrain boundary misorientation	°	5	This work
$f_{r}$	Material coefficient for subgrain refinement	-	$3.5\cdot 10^{9}[{\dot{\varepsilon }exp(Q/RT)]}^{-0.23}$	This work
$f_{c}$	Material coefficient for subgrain coarsening	-	$6000\;[{\dot{\varepsilon }exp(Q/RT)]}^{0.53}$	This work
A	A parameter	-	$2.76\;exp\left(0.0046T\right)$	This work
B	B parameter	-	2.5	This work
C	C parameter	-	1·10^−3^	This work

## Data Availability

The raw data supporting the conclusions of this article will be made available by the authors on request.

## References

[1] Humphreys F.J., Hatherly M. (2004). Recrystallization and Related Annealing Phenomena.

[2] Doherty R.D., Hughes D.A., Humphreys F.J., Jonas J.J., Jensen D.J., Kassner M.E., McNelley T.R., McQueen H.J., Rollet A.D. (1997). Current issues in recrystallization: A review. Mater. Sci. Eng. A.

[3] Lv J.X., Zheng J.H., Yardley V.A., Shi Z.S., Lin J.G. (2020). A review of microstructural evolution and modelling of aluminium alloys under hot forming conditions. Metals.

[4] Kaibyshev R., Shipilova K., Musin F., Motohashi Y. (2005). Continuous dynamic recrystallization in an Al-Li-Mg-Sc alloy during equal-channel angular extrusion. Mater. Sci. Eng. A.

[5] Li J.C., Wu X.D., Cao L.F., Liao B., Wang Y.C., Liu Q. (2021). Hot deformation and dynamic recrystallization in Al-Mg-Si alloy. Mater. Charact..

[6] Lee J.W., Son K.T., Jung T.K., Yoon Y.O., Kim S.K., Choi H.J., Hyun S.K. (2016). Continuous dynamic recrystallization behavior and kinetics of Al-Mg-Si alloy modified with CaO-added Mg. Mater. Sci. Eng. A.

[7] Feng X.M., Wang Y.T., Huang Q.G., Liu H.L., Zhang Z.H. (2024). The dynamic recrystallization microstructure characteristics and the effects on static recrystallization and mechanical properties of Al–Mg–Si alloy. Mater. Sci. Eng. A.

[8] Zhang J.J., Yi Y.P., Huang S.Q., Mao X.C., He H.L., Tang J.G., Guo W.F., Dong F. (2021). Dynamic recrystallization mechanisms of 2195 aluminum alloy during medium/high temperature compression deformation. Mater. Sci. Eng. A.

[9] Sakai T., Miura H., Goloborodko A., Sitdikov O. (2009). Continuous dynamic recrystallization during the transient severe deformation of aluminum alloy 7475. Acta Mater..

[10] Maizza G., Pero R., Richetta M., Montanari R. (2018). Continuous dynamic recrystallization (CDRX) model for aluminum alloys. J. Mater. Sci..

[11] Yang Q., Wojcik T., Kozeschnik E. (2024). Continuous dynamic recrystallization and deformation behavior of an AA1050 aluminum alloy during high-temperature compression. Metals.

[12] Huang K., Logé R.E. (2016). A review of dynamic recrystallization phenomena in metallic materials. Mater. Des..

[13] Sakai T., Belyakov A., Kaibyshev R., Miura H., Jonas J.J. (2014). Dynamic and post-dynamic recrystallization under hot, cold and severe plastic deformation conditions. Prog. Mater. Sci..

[14] Zhang J.J., Yi Y.P., He H.L., Huang S.Q., Mao X.C., Guo W.F., You W., Guo Y.L., Dong F., Tang J.G. (2021). Kinetic model for describing continuous and discontinuous dynamic recrystallization behaviors of 2195 aluminum alloy during hot deformation. Mater. Charact..

[15] Ding S., Khan S.A., Yanagimoto J. (2020). Flow behavior and dynamic recrystallization mechanism of A5083 aluminum alloys with different initial microstructures during hot compression. Mater. Sci. Eng. A.

[16] Li H., Huang Y.C., Liu Y. (2023). Dynamic recrystallization mechanisms of as-forged Al–Zn–Mg-(Cu) aluminum alloy during hot compression deformation. Mater. Sci. Eng. A.

[17] Illgen C., Bohne B., Wagner M.F.X., Frint P. (2022). Thermal stability of SPD-processed aluminum alloys—Internal friction as an indication for recovery, recrystallization and abnormal grain growth. J. Mater. Res. Technol..

[18] Meng Z.J., Zhang C.S., Zhang G.F., Wang K.Z., Wang Z.J., Chen L., Zhao G.Q. (2023). Hot compressive deformation behavior and microstructural evolution of the spray-formed 1420 Al–Li alloy. J. Mater. Res. Technol..

[19] Wang K.Z., Zhang C.S., Cheng Z.N., Zhao H.B., Meng Z.J., Chen L., Zhao G.Q. (2024). Dynamic evolution of the T1 phase and its effect on continuous dynamic recrystallization in Al–Cu–Li alloys. Int. J. Plast..

[20] Velay X. (2009). Prediction and control of subgrain size in the hot extrusion of aluminium alloys with feeder plates. J. Mater. Process. Technol..

[21] Poletti C., Rodriguez-Hortalá M., Hauser M., Sommitsch C. (2011). Microstructure development in hot deformed AA6082. Mater. Sci. Eng. A.

[22] Sellars C., Zhu Q. (2000). Microstructural modelling of aluminium alloys during thermomechanical processing. Mater. Sci. Eng. A.

[23] Furu T., Ørsund R., Nes E. (1995). Subgrain growth in heavily deformed aluminium-experimental investigation and modelling treatment. Acta Metall. Mater..

[24] Nes E., Vatne H.E., Daaland O., Furu T., Ørsund R., Marthinsen K. (1994). Physical Modelling of Microstructural Evolution during Thermomechanical Processing of Aluminium Alloys.

[25] Nes E. (1998). Modelling of work hardening and stress saturation in FCC metals. Prog. Mater. Sci..

[26] Sheppard T. (1996). Development of structure, recrystallization kinetics and prediction of recrystallized layer thickness in some Al-alloys. Proceedings of the 6th International Seminar on Aluminium Extrusion Technology.

[27] Duan X., Sheppard T. (2002). Simulation of substructural strengthening in hot flat rolling. J. Mater. Process. Technol..

[28] Duan X., Sheppard T. (2002). Three dimensional thermal mechanical coupled simulation during hot rolling of aluminium alloy 3003. J. Mater. Sci..

[29] Marthinsen K., Nes E. (2001). Modelling strain hardening and steady state deformation of Al-Mg alloys. Mater. Sci. Technol..

[30] Gourdet S., Montheillet F. (2003). A model of continuous dynamic recrystallization. Acta Mater..

[31] Sun Z.C., Wu H.L., Cao J., Yin Z.K. (2018). Modeling of continuous dynamic recrystallization of Al-Zn-Cu-Mg alloy during hot deformation based on the internal-state-variable (ISV) method. Int. J. Plast..

[32] Chen S.F., Li D.Y., Zhang S.H., Han H.N., Lee H.W., Lee M.G. (2020). Modelling continuous dynamic recrystallization of aluminum alloys based on the polycrystal plasticity approach. Int. J. Plast..

[33] Chen F., Tian X., Wu G., Zhu H., Ou H., Cui Z. (2022). Coupled quantitative modeling of microstructural evolution and plastic flow during continuous dynamic recrystallization. Int. J. Plast..

[34] Furu T., Shercliff H.R., Sellars C.M., Ashby M.F. (1996). Physically-based modelling of strength, microstructure and recrystallisation during thermomechanical processing of Al-Mg alloys. Mater. Sci. Forum.

[35] Sherby O.D., Klundt R.H., Miller A.K. (1977). Flow stress, subgrain size, and subgrain stability at elevated temperature. Metall. Trans. A.

[36] Hallberg H., Wallin M., Ristinmaa M. (2010). Modeling of continuous dynamic recrystallization in commercial-purity aluminum. Mater. Sci. Eng. A.

[37] Huang Y., Humphreys F.J. (2000). Subgrain growth and low angle boundary mobility in aluminium crystals of orientation {110}〈001〉. Acta Mater..

[38] Sherstnev P., Lang P., Kozeschnik E. Treatment of simultaneous deformation and solid-state precipitation in thermo-kinetic calculations. Proceedings of the 6th European Congress on Computational Methods in Applied Sciences and Engineering (ECCOMAS 2012).

[39] Kreyca J., Kozeschnik E. (2018). State parameter-based constitutive modelling of stress strain curves in Al-Mg solid solutions. Int. J. Plast..

[40] Buken H., Kozeschnik E. (2021). Modeling static recrystallization in Al-Mg alloys. Metall. Mater. Trans. A.

[41] Raabe D., Laughlin D.E., Hono K. (2014). 23—Recovery and Recrystallization: Phenomena, Physics, Models, Simulation. Physical Metallurgy.

[42] McQueen H.J., Kassner M.E. (2004). Comments on ‘a model of continuous dynamic recrystallization’ proposed for aluminum. Scr. Mater..

[43] Federighi T. (1959). A possible determination of the activation energy for self-diffusion in aluminium. Philos. Mag..

[44] Hirth J.P., Lothe J. (1991). Theory of Dislocations.

[45] Galindo-Nava E.I., Rivera-Díaz-del-Castillo P.E.J. (2012). A thermostatistical theory of low and high temperature deformation in metals. Mater. Sci. Eng. A.

[46] Mecking H., Nicklas B., Zarubova N. (1986). A universal temperature scale for plastic flow. Acta Mater..

[47] Frost H., Ashby M. (1982). Deformation-Mechanism Maps.

[48] Bergström Y. (1983). The plastic deformation of metals-a dislocation model and its applicability. Rev. Powder Metall. Phys. Ceram..

[49] Alankar A., Field D.P., Raabe D. (2014). Plastic anisotropy of electro-deposited pure a-iron with sharpcrystallographic <111>// texture in normal direction: Analysis by an explicitly dislocation-based crystal plasticity model. Int. J. Plast..

[50] Mirzadeh H., Cabrera J.M., Najafizadeh A. (2012). Modeling and prediction of hot deformation flow curves. Metall. Mater. Trans. A.

[51] Gourdet S., Montheillet F. (2000). An experimental study of the recrystallization mechanism during hot deformation of aluminium. Mater. Sci. Eng. A.

[52] Yanushkevich Z., Belyakov A., Kaibyshev R. (2015). Microstructural evolution of a 304-type austenitic stainless steel during rolling at temperatures of 773-1273 K. Acta Mater..

[53] Wu G.C., Lin Y.C., Chen M.S., Qiu W., Zeng N.F., Zhang S., Wan M., He D.G., Jiang Y.Q., Naseri M. (2024). Continuous dynamic recrystallization behaviors in a single-phase deformed Ti-55511 alloy by cellular automata model. J. Alloys Compd..

[54] Hines J.A., Vecchio K.S., Ahzi S. (1998). A model for microstructure evolution in adiabatic shear bands. Metall. Mater. Trans. A.

[55] Wu S., Fan K., Jiang P., Chen S. (2010). Grain refinement of pure Ti during plastic deformation. Mater. Sci. Eng. A.

[56] Hansen N. (2004). Hall–Petch relation and boundary strengthening. Scr. Mater..

[57] Summers P.T., Mouritz A.P., Case S.W., Lattimer B.Y. (2015). Microstructure-based modeling of residual yield strength and strain hardening after fire exposure of aluminum alloy 5083-H116. Mater. Sci. Eng. A.

